# Clinical Significance of Annexin A4 as a Biomarker in the Early Diagnosis of Hepatocellular Carcinoma

**DOI:** 10.31557/APJCP.2020.21.9.2661

**Published:** 2020-09

**Authors:** Zienab M Saad, Yasser Fouad, Lamia H Ali, Taha M Hassanin

**Affiliations:** 1 *Endemic Medicine Department, Faculty of Medicine, Minia University, Minia, Egypt.*; 2 *Clinical Pathology Department, Faculty of Medicine, Minia University, Minia, Egypt. *

**Keywords:** Hepatocellular carcinoma, hepatitis C virus, tumor markers, Annexin A4, Alpha-fetoprotein

## Abstract

**Background:**

Hepatocellular carcinoma (HCC) is one of the most prevalent cancer worldwide. Early detection of HCC is crucial to improve prognosis and survival. Nearly 30 % of HCC patients present with normal serum alpha fetoprotein (AFP), which highlights the need for new biomarkers for HCC. Annexin A4 (ANXA_4_) is one of the annexin family with high expressions found in gastric, liver, lung, colorectal and ovarian cancers. Aim : to evaluate the clinical significance of ANXA_4_ in the early diagnosis of HCC.

**Methods:**

Thirty patients with hepatitis C virus (HCV) related HCC were enrolled in this study. They were stage A according to Barcelona Clinic Liver Cancer (BCLC) staging and they were grade A or B according to Child Pugh Classification. Twenty patients with HCV-related liver cirrhosis and 20 healthy persons seronegative for both HCV and HBV served as control group. ANXA_4_ and AFP were measured in serum of all cases.

**Results:**

Serum ANXA_4_ level was significantly higher in HCC patients compared to patients with liver cirrhosis and healthy controls (188, IQR 42-428 and 23, IQR 24-33 and and 21, IQR 22-24 ng / ml, respectively). By using the ROC curve, the area under the curve of ANXA_4_ was 0.972 and the best cut-off value was115 ng/ml, with sensitivity 95% and specificity 80%.

**Conclusion:**

The serum level of ANXA_4_ might be a good biomarker for the early detection of HCC.

## Introduction

Hepatocellular carcinoma (HCC) is the 5^th^ common cancer in men however the 7^th^ in women and the 3^rd ^leading cause of cancer-related deaths worldwide (Jemal et al., 2011). Liver resection might be the first choice for the treatment of patients with intermediate-stage HCC due to its effects on reducing HCC recurrence and improving long-term quality of life (Xie et al., 2015). In addition, radiofrequency ablation and liver transplantation are potential curative methods for HCC (Thomas and Zhu, 2005). Most HCC patients are diagnosed at late stage with chronic underlying liver dysfunction, which makes curative treatments not feasible (Bosch et al., 1999). About 10 – 20 % of tumors are resectable at the time of diagnosis, and the 5-year survival is poor even when compared with other gastrointestinal malignancies (El-Serag, 2020). Therefore, it is of great importance to find a biomarker which can be used for early diagnosis of HCC and hence, can improve prognosis and treatment efficiency.

Annexin A4 (ANXA_4_) is one of the annexin family, which first discovered in animal cells and were named for their ability to “annex” or aggregate membranes (Lokman et al., 2011; Bharadwaj et al., 2013). The ANXA_4_ was found to have important functions in membrane permeability, exocytosis, and regulation of chloride conductance (Sohma et al., 2001; Hill et al., 2003). Also, ANXA_4_ has been reported to be involved in tumor spread and anti-cancer drug resistance (Mussunoor and Murray, 2008 ), and was identified as a potential biomarker for gall bladder cancer (Huang et al., 2014). ANXA_4_ expression has been associated with loss of cell-to-cell adhesion, increased metastasis, and chemo-resistance, and therefore is regarded as a potential diagnostic and therapeutic marker in cancer field (Kim et al., 2009). Moreover, ANXA_4_ was found to be upregulated in human primary HCC tissues, promotes HCC cell proliferation, and ANXA_4_ knockdown decreased the tumorigenic potential in a subcutaneous xenograft model and that ANXA_4_ is crucial for HCC proliferation (Liu et al., 2017). This study was designed to evaluate the clinical significance and potential role of ANXA_4_ in the early diagnosis of HCC.

Patients and methods: The study was approved by the institutional review board of Minia University Faculty of Medicine, and in accordance with the ethical standards of Clinical Research Committee of Minia University Hospital. Written, informed consent was obtained from every participant. It is a prospective cross sectional study that included three patient groups. Group 1: Thirty patients with early stage HCC on top of liver cirrhosis, diagnosed according to the Barcelona Clinic Liver Cancer staging system (BCLC-A) (single or 3 nodules < 3 cm; `Performance Status 0) (Llovet et al., 2004). The diagnosis of HCC was based on imaging studies (abdominal ultrasound, CT, or MRI) and AFP serology. Group 2: Twenty patients with liver cirrhosis (LC), in whom diagnosis of liver cirrhosis was based on clinical examination, laboratory findings and ultrasonographic findings suggestive of LC. Group 3: twenty age and sex-matched healthy persons were enrolled as a control group. The healthy controls were collected from the healthy volunteer blood donors, in whom liver and other systemic diseases were excluded by history, physical examination, laboratory and ultrasonographic assessment. Group 1 and 2 patients were recruited consecutively from liver cirrhosis patients who attended the HCV-Clinic at Minia University Hospital for their regular medical follow up. All participants of the 3 groups were subjected to thorough history taking, clinical examination, abdominal ultrasonography, laboratory investigations including serum albumin, serum bilirubin, alanine and aspartate transaminase serum urea and creatinine, complete blood count, prothrombin time, and blood sugar level. Exclusion criteria for HCC group: 1. intermediate or advanced stage HCC as defined by the BCLC staging system; 2. Portal vein thrombosis; 3. Local or distant metastasis as confirmed by radiological imaging studies; 4. patients with other systemic disease i.e, chronic renal, chest, or heart disease. All participants were tested for hepatitis B surface antigen and hepatitis C virus antibodies and HCV RNA in HCV antibody-positive patients. Blood samples were withdrawn from each participant under complete aseptic conditions for the routine laboratory investigations, serum AFP detection, and serum level of ANXA_4_. Serum ANXA_4_ levels were measured by enzyme-linked immunosorbent assay (ELISA) with a commercially available ELISA kit (supplied by immunoassay kit, Gen Asia Co., China) according to the manufacturer’s directions. The level of ANXA_4_ protein was obtained through standard curve. Results were reported as concentration of ANXA_4_ ng/ml in samples. 

Statistical analysis: Statistical analyses were performed using SPSS 20 and Graph Pad Prism 5 (Graph Pad Software Inc., CA, USA). Numerical variables were recorded as either means ± SD, or median and IQR, and analyzed by Wilcoxon test. Categorical variables were presented as number and percentages and analyzed using the Chi-square test. The correlation between ANXA_4_ concentrations in serum and AFP was analyzed with Pearson’s* χ*^2^ test. The diagnostic accuracy of ANXA_4_ and AFP was determined by a receiver operating characteristic (ROC) curve analysis. The diagnostic cut-off, the related sensitivity and specificity, and the positive and negative predictive values (PPV, NPV) were also determined. A p value < 0.05 was considered significant.

## Results

**Figure 1:**
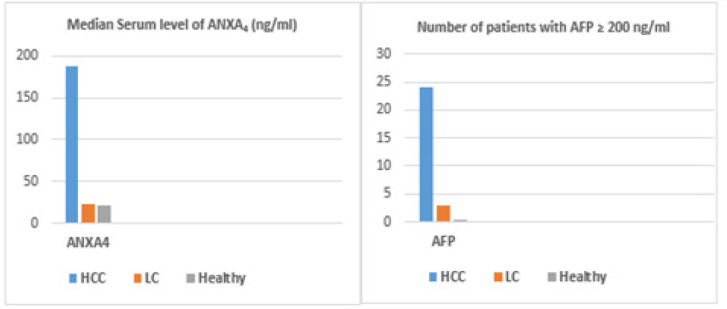
Comparison between the 3 Studied Groups Regarding Serum Level of ANXA_4_ and AFP

**Figure 2:**
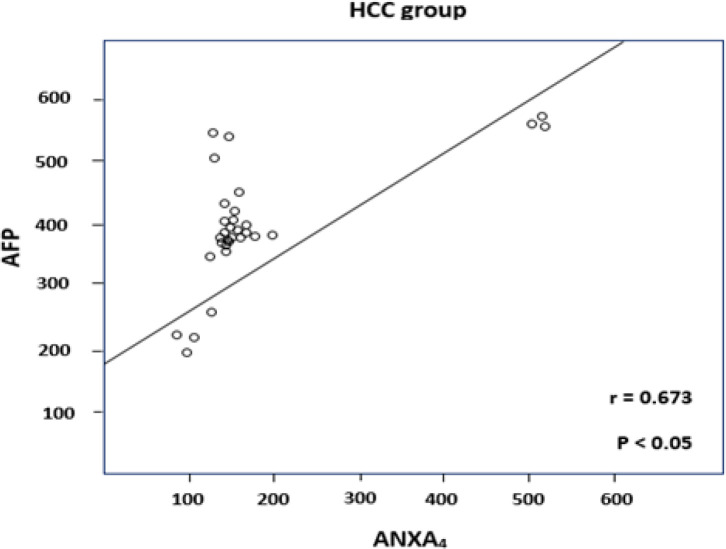
Correlation between Serum Level of AFP and ANXA_4_ in HCC Group

**Figure 3.**
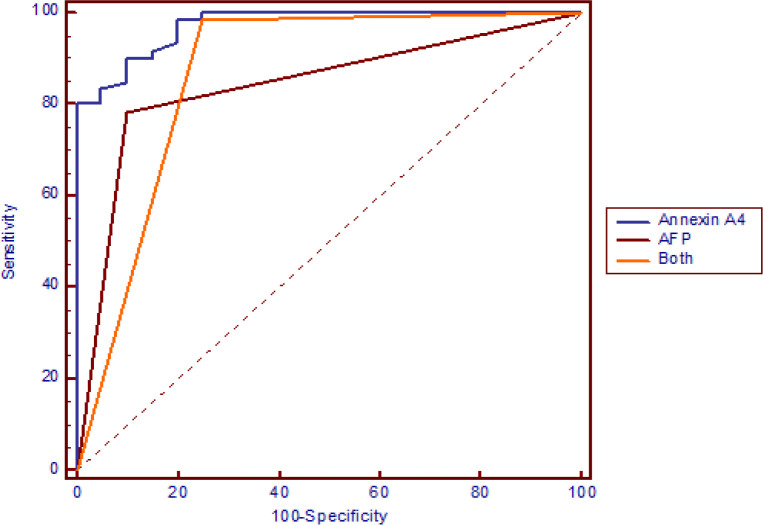
Receiver Operating Characteristic Curve (ROC) Analysis Shows the Diagnostic Performance of ANXA_4 _and AFP in Discriminating Patients with Hepatocellular Carcinoma from Those with Liver Cirrhosis

The demographic features of the 3 studied groups were as follows: HCC group: 24 males (80%) and 6 females (20%) with an age range from 46 to 65 years, and LC group: 15 males (75%) and 5 females (25%) with an age range from 38 to 61 years. Twenty healthy subjects: 14 males (70%) and 6 females (30%) with an age range between 36 and 45 years (healthy control group). Descriptive statistics of laboratory parameters of the 3 studied groups are shown in Table 1 and Figure 1. Comparative statistics between serum levels of AFP and ANXA_4_ in the 3 studied groups are shown in Table 2: regarding to ANXA_4_, it showed a significantly higher levels in patients with HCC compared with patients with LC (p < 0.05) and compared with controls (p < 0.05). However, the level of ANXA_4_ was not significantly different in patients with LC compared with the control group (p > 0.05). Regarding to AFP, it showed a significant differences between patients with HCC compared with patients with LC (p < 0.05) and compared with controls (p < 0.05). Also, a significant difference was observed between patients with LC and controls (p < 0.05). In addition, we found a significant correlation between the serum level of ANXA_4_ and that of alpha fetoprotein in the HCC group as shown in Figure 2. ROC curve analysis was performed to assess the diagnostic performance of ANXA_4_ in discriminating patients with HCC from those with liver cirrhosis. This analysis revealed that the best cut-off value for ANXA_4_ was 115 ng/dl with a sensitivity and specificity 95 %, and 80%, respectively, and with a positive predictive value (PPV) of 93 %, and negative predictive value (NPV) of 94%. While the sensitivity and specificity of AFP at cut off value ≥ 200 ng/ml was 78 % and 90%, respectively, and the PPV was 95 % and the NPV was 58 % as shown in Figure 3 and Table 3.

**Table 1:** Descriptive Statistical Data of Various Parameters in the 3 Studied Groups

	group I	Group II	group III
	HCC (n=30)	liver cirrhosis (n=20)	Healthy Control (n=20)
HG (gm/dl) Mean ± SD	8.16 ±1.5	8.6 ± 0.7	12.6 ± 0.7
Platelets (x10^3^/mm^3^) Mean ± SD	103.1 ± 15.9	113.3 ±1 2.5	199.4 ± 23.9
ALT (U/l) Mean ± SD	61.2 ± 18.7	65.9 ± 20.8	27.1 ± 3.8
AST (U/l) Mean ± SD	66.9 ± 19.5	69.7 ± 20.2	28.2 ± 5.3
T. Bil (mg/dl) Mean ± SD	3.3 ± 0.8	3.2 ± 0.9	0.9 ± 0.1
Creatinine (mg/dl) Mean ± SD	1.8 ± 0.9	1.7 ± 0.9	0.9 ± 0.3
INR: Mean ± SD	1.3±0.5	1.2 ± 0.6	0.9 ± 0.2
Albumin (gm/dl) Mean ± SD	2.5 ± 0.4	2.6 ± 0.3	4.1 ± 0.4
ANXA4 (ng/ml)Median	188	23	21
IQR	(42 - 428)	(24 - 33)	(22 - 24)
AFP < 200 ng/ml (No of patients)	6 (20%)	17 (85%)	20 (100%)
AFP ≥ 200 ng/ml (No of patients)	24 (80 %)	3 (15%)	0 (0%)

HG, hemoglobin; IQR, inter quartile ratio; AFP, alpha fetoprotein; No, number; HCC, Hepatocellular carcinoma; T. Bil, total bilirubin; AST, aspartate transaminase; ALT, alanine transaminase; INR, international normalization ratio

**Table 2:** Statistical Comparison between ANXA_4_ and AFP in the 3 Studied Groups

Variable	HCC Vs LC	HCC Vs control	LC Vs control
	*P*-value	*P*-value	*P*-value
ANXA_4_ (ng/ml)^1^	< 0.05*	< 0.05*	> 0.05
AFP ( > 200 ng/ml)^2^	< 0.05*	< 0.05*	< 0.05*

*, statistically significant; AFP, alpha-fetoprotein; ANXA_4_, Annexin 4; HCC, Hepatocellular carcinoma; LC, liver cirrhosis.

**Table 3:** ROC Curve Analysis of ANXA_4_ and AFP for Prediction of HCC in Cirrhotic Patients

Variable	Optimal cutoff	AUC	Sensitivity	Specificity	PPV	NPV	Accuracy
ANXA_4_ (ng/ml)	≥ 115	0.972	95%	80%	93%	94%	93.50%
AFP (ng/ml)	≥ 200	0.842	78%	90%	95%	58%	81.50%
ANXA_4_ & AFP			98%	88%	92%	93%	93%

ANXA_4_, Annexin A4; AFP, alpha-fetoprotein; AUC, area under the curve; PPV, positive predictive value; NPV, negative predictive value

## Discussion

Egypt is a country with high HCV prevalence (El-Ghitany, 2019). The diagnosis of HCC is challenging owing to the high prevalence of HCV infection and HCV-related liver cirrhosis and the high percentage of patients who develop HCC (Shaker et al., 2013). Imaging studies cannot always differentiate between benign hepatic lesions and HCC. Also, the imaging based diagnosis of small tumors is relatively inaccurate as dysplastic nodules or fibro nodules can resemble HCC (Bolog et al., 2011). Moreover, the lack of equipment and the high cost of abdominal CT and/or MRI (especially in the developing countries) make the early diagnosis of HCC difficult. In addition, the traditional liver function tests do not distinguish HCC from liver cirrhosis (Thapa and Walia, 2007) Accordingly, there is strong need to a serum marker which is simple, accurate and cost-effective in the diagnosis of HCC, but unfortunately it is not found yet. Serum AFP has been regarded as the most useful serum marker for patients at risk for HCC. However, its sensitivity for the detection of HCC is low ranging from 40% to 65%, and its specificity is variable (80 - 94%). High serum AFP level can be seen in patients with liver cirrhosis (up to 47%) and chronic active hepatitis (up to 58%). Additionally, a small hepatic tumor may result in AFP level lower than the limit of detection, whereas AFP level may be delayed in some large hepatic tumors yielding AFP-negative HCC (Zhao et al., 2013; Zhu et al, 2013 ). For these reasons, there are ongoing and continuous studies to determine a sensitive and specific new diagnostic markers for HCC. There is few data available about serum level of ANXA_4_ in patients with HCC and the aim of this study was to evaluate the serum level of ANXA_4_ as a diagnostic marker of HCC and compare it with that of AFP. Chen et al., (2016), found that ANXA_4_ is significantly elevated in HCC patients with early recurrence, and had a strong correlation with portal vein tumor thrombosis (p = 0.03) and advanced BCLC stage (p = 0.002) and might act as a potential prognostic biomarker for HCC. They reported that annexin A4 might facilitate HCC cell migration and invasion via regulating epithelial-mesenchymal transition and they concluded that patients with high levels of ANXA_4_ had higher recurrence rate and shorter overall survival than those with low expression. Our results showed a significantly higher serum levels of AFP in patients with HCC compared with LC patients and compared with healthy controls; and this is going with that of El-Tayeh et al, 2012. Also, we found a significantly higher serum level of AFP in patients with LC compared to healthy controls; and this is in agreement with that of Page et al, (2014), who reported that AFP might increase in patients with hepatitis and liver cirrhosis without HCC. It was stated that hepatic injury; such as during active hepatitis C virus infection; can increase the serum levels of AFP in patients who do not have HCC (El-Serag, 2020). Regarding to ANXA_4_, our study showed that ANXA_4_ level was significantly higher in patients with early HCC compared with cirrhotic patients (p < 0.05) and with healthy controls (p < 0.05). We used ROC curve analysis to assess the clinical utility of ANXA_4_ and AFP in discriminating patients with HCC from those with liver cirrhosis which revealed that the area under the curve (AUC) for ANXA_4_ was (0.972), and the best cut off value was115 ng/ml with a sensitivity 95% and a specificity 80%, PPV 93% and NPV 94%, while the AUC for AFP was (0.842) and the best cut off value was 200 ng/ml with a sensitivity 78%, a specificity 90%, PPV 95% and NPV 58%. This means that ANXA_4_ is more sensitive but less specific than AFP. Both ANXA_4_ and AFP have comparable PPV but ANXA_4_ has better NPV. Combining both ANXA_4_ and AFP has increased the diagnostic efficiency of AFP as shown in Table 3. 

In conclusion, this study showed that the serum level of ANXA_4_ might be a good biomarker for the early detection of HCC since it has a higher sensitivity than AFP. Also, we found that ANXA_4_ can differentiate between HCC and liver cirrhosis since we found that the levels of ANXA_4_ were significantly higher in patients with HCC than in cirrhotic patients. The main study limitations are: the study included only patients with HCV related HCC, and the small number of the included patients. Further studies on a larger patient scale in the future is recommended. 

## Author contributions

Yasser M Fouad, Zienab M Saad and Taha M Hassanin designed the study and drafted the manuscript.

Taha M Hassanin and Zienab M Saad examined the patients and collect the data.

Lamia H Ali carried out the lab part of the work.

Yasser M Fouad, Taha M Hassanin and Zienab M Saad analyzed and interpreted the data.

All authors read, wrote, and approved the final manuscript.

## Conflict-of-interest statement

None of the authors have any conflicts of interest or financial disclosures.
